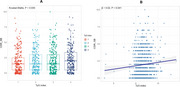# Insulin resistance exacerbates cognitive impairment in patients with Alzheimer's disease

**DOI:** 10.1002/alz70856_106611

**Published:** 2026-01-09

**Authors:** Shiyu Feng, Hanlin Cai, Ruihan Wang, Hui Gao, Yingying Tang, Linyuan Qin, Caimei Luo, YiMeng Ren, Feng Yang, Mengyao Guo, Qian Liao, Dong Zhou, Qin Chen

**Affiliations:** ^1^ West China Hospital of Sichuan University, Chengdu, Sichuan, China

## Abstract

**Background:**

Insulin resistance, a manifestation of insulin signaling pathway disorders, can induce neuroinflammation by interacting with Alzheimer's disease (AD) pathology. The triglyceride‐glucose (TyG) index provides an effective method to reflect insulin resistance levels. However, the role of insulin resistance in the pathophysiology of AD remains unclear. This study aimed to investigate the association between TyG index and cognitive function in patients with AD.

**Method:**

A total of 1029 participants with positive β‐amyloid biomarkers were included from the Alzheimer's Disease Neuroimaging Initiative (ADNI), according to the amyloid PET or cerebrospinal fluid Aβ42 levels. Triglyceride‐glucose (TyG) index was calculated using fasting blood glucose and triglyceride levels. Comprehensive cognitive level was represented by the global Clinical Dementia Scale (CDR‐G), CDR‐sum of boxes (CDR‐SB), Mini‐Mental State Examination (MMSE), and Preclinical Alzheimer's Cognitive Composite (PACC). Multivariate linear regression analysis was used to examine the association between TyG index and cognitive function, adjusting for age, gender, education, race, marriage, smoking and drinking history, apolipoprotein E genotype, BMI, hypertension and diabetes history.

**Result:**

Patients with AD in the highest TyG quartile group demonstrated the worst Clinical Dementia Rating‐Sum of Boxes (CDR‐SB) scores (*p* = 0.035). A higher TyG index was correlated with higher CDR‐SB (r=0.065, *p* = 0.035), lower PACC (r=‐0.048, *p* = 0.022), and lower MMSE (r=‐0.052, *p* = 0.018) scores. Multivariate regression analysis confirmed that TyG index remained independently associated with higher CDR‐SB scores (β = 0.02, 95% CI = [0.01, 0.04], *p* =  0.041) after adjusting for age, gender, education, race, marriage, smoking and drinking history, apolipoprotein E genotype, BMI, hypertension and diabetes history.

**Conclusion:**

Our findings indicate a significant association between insulin resistance and cognitive impairment in patients with Alzheimer's disease, suggesting the potential importance of insulin resistance in AD pathophysiology.